# Effect of Poly(propylene carbonate) on Properties of Polylactic Acid-Based Composite Films

**DOI:** 10.3390/ijms25094730

**Published:** 2024-04-26

**Authors:** Kang Chen, Xinyu Zhang, Zanru Wang, Ce Sun, Haiyan Tan, Yanhua Zhang

**Affiliations:** 1 Key Laboratory of Bio-Based Material Science and Technology, Northeast Forestry University, Ministry of Education, Harbin 150040, China; chenk0777@163.com (K.C.); 317855088@nefu.edu.cn (X.Z.); tanhaiyan@nefu.edu.cn (H.T.); 2 School of Mechanical and Power Engineering, Harbin University of Science and Technology, Harbin 150080, China; 18742039230@163.com

**Keywords:** composite film, degradability, polylactic acid, poly(propylene carbonate), thermal analysis

## Abstract

To enrich the properties of polylactic acid (PLA)-based composite films and improve the base degradability, in this study, a certain amount of poly(propylene carbonate) (PPC) was added to PLA-based composite films, and PLA/PPC-based composite films were prepared by melt blending and hot-press molding. The effects of the introduction of PPC on the composite films were analyzed through in-depth studies on mechanical properties, water vapor and oxygen transmission rates, thermal analysis, compost degradability, and bacterial inhibition properties of the composite films. When the introduction ratio coefficient of PPC was 30%, the tensile strength of the composite film increased by 19.68%, the water vapor transmission coefficient decreased by 14.43%, and the oxygen transmission coefficient decreased by 18.31% compared to that of the composite film without PPC, the cold crystallization temperature of the composite film increased gradually from 96.9 °C to 104.8 °C, and PPC improved the crystallization ability of composite film. The degradation rate of the composite film with PPC increased significantly compared to the previous one, and the degradation rate increased with the increase in the PPC content. The degradation rate was 49.85% and 46.22% faster on average than that of the composite film without PPC when the degradation was carried out over 40 and 80 days; the composite film had certain inhibition, and the maximum diameter of the inhibition circle was 2.42 cm. This study provides a strategy for the development of PLA-based biodegradable laminates, which can promote the application of PLA-based laminates in food packaging.

## 1. Introduction

Poly(propylene carbonate) (PPC) is a biodegradable aliphatic polycarbonate made by copolymerizing carbon dioxide (CO_2_) and propylene oxide (PO) alternately in the presence of a catalyst [1,2]. The capture and consumption of CO_2_ in the synthesis process offer the possibility to reduce the greenhouse effect and mitigate oil shortages [3,4]. PPC has attracted much attention for its excellent biodegradability, biocompatibility, and gas barrier properties, thus showing great potential for applications in food packaging, disposable tableware, and biomedical materials [5,6]. However, due to the amorphous structure and low-glass transition temperature of PPC, PPC has the disadvantages of high brittleness at low temperatures and poor strength at high temperatures, and its poor stability and low mechanical strength seriously limit the scope of its application [7,8,9].

In order to expand the application of PPC, many scholars have conducted a series of studies on PPC and found that an economical and promising method to improve the mechanical and thermal properties of PPC is to mix PPC with other biodegradable polymers [10,11,12,13]. Among them, polylactic acid (PLA) has the advantages of high strength, high modulus, and excellent processing performance [14,15], and the chemical structure of PPC and PLA is similar, so PPC and PLA have a certain degree of compatibility when blending, and the two can provide a complementary performance [9,16,17]. By melt blending PPC with PLA, composites with better overall performance can be obtained [18,19]. The composting degradability of PPC/PLA composites is much better than that of PLA, and the melt blending process is simple and easy.

As a result, researchers began to investigate the preparation of composites by mixing PPC with PLA. Park et al. [20] found two yield plateaus in the stress–strain curves of PLA/PPC blends when the addition of PLA was in the range of 30–70%, and the fracture energy of PLA/PPC blends was higher. Wang et al. [21] prepared composites using a blend of PPC, PLA, and montmorillonite (OMMT) and found that the tensile strength of the composites could reach up to 20.83 MPa, which was 12.1 and 1.85 times higher than that of PPC and PPC/PLA, respectively. Yang et al. [22] directly melt-blended PPC with 10 wt% PLA and 1.5 wt% multi-walled carbon nanotubes (CNTs) and found that the mechanical strength of PPC could be significantly improved by adding low levels of both PLA and CNTs without losing its good ductility. Its mechanical properties are comparable to or even better than those of traditional petroleum-based polymers, such as low-density polyethylene (LDPE), high-density polyethylene (HDPE), polypropylene (PP), and polystyrene (PS). Lin et al. [23] prepared composites consisting of PPC, PLA, and seafoam nanofibers by a two-step melt mixing and annealing process and verified them by rheological and morphological characterization. The results showed that the mechanical and thermal properties of PPC were greatly improved, and the modulus of elasticity of the composites at 100 °C was about three orders higher than that of pure PPC. Cvek et al. [24] added curcumin (CCM) to PLA/PCC samples to effectively absorb UV radiation while maintaining film transparency (T_700_∼68–84%). The CCM extract in ethanol was studied with 1,1-diphenyl-2-picrylhydrazyl (DPPH), and analyses showed that the samples also provided effective antioxidant effects due to the tunable release of CCM. Water vapor and oxygen permeability analyses showed that PPC improved the barrier properties of PLA/PPC blends, whereas the presence of CCM did not hinder the barrier properties. Hao et al. [25] modified PLA/PPC composites using a flame-retardant guanidine phosphate (GP). It was concluded that GP contributed to the enhancement of the glass transition temperature T_g_ and flame retardancy of the composites. In total, 3 wt% of GP enabled the PLA/PPC composites to achieve T_g_ of 60.0 °C, a UL-94 rating of V-0, and a limiting oxygen index (LOI) of 30.8%.

In this study, PLA/PPC composite films were prepared by adding a certain amount of PPC to PLA, and the composite films were modified by the introduction of poly(butyleneadipate-co-terephthalate) (PBAT), chitosan (CS) and the chain extender ADR. The mechanical properties, water vapor and oxygen barrier, thermal stability, degradation properties, and antibacterial properties of the composite films were tested by melt blending and hot pressing. The presence of the epoxy chain extender ADR in the composite film effectively improved the intermolecular interactions between PLA, PBAT, PPC, and CS, resulting in better compatibility of the obtained composite film. CS has good antibacterial properties, biodegradability, and biocompatibility, which can improve the functionality of laminated films in the food packaging field.

## 2. Results and Discussion

### 2.1. Analysis of Structural Properties of Composite Film

**Figure 1:**
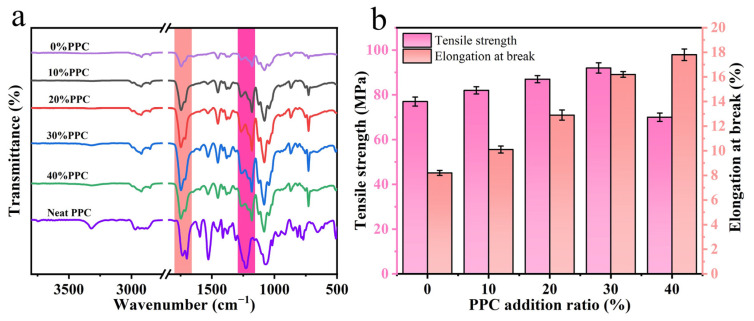
FTIR of composite films and PPC (**a**), tensile strength and elongation at break of composite films (**b**).

**Figure 2:**
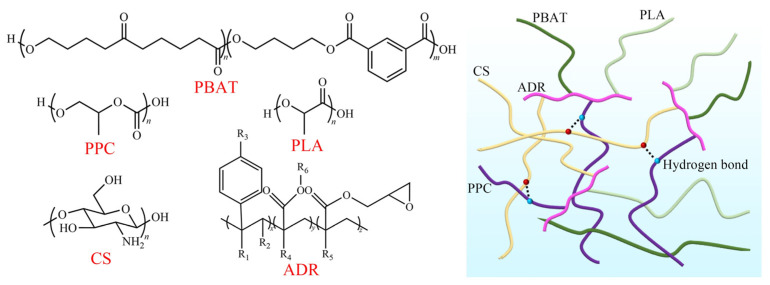
Chemical structure and binding mechanism of each component of composite films.

The FTIR infrared spectra of the composite film and PPC were detected, as shown in Figure 1a. The shift or broadening of the absorption bands in the FTIR spectra indicated the presence of significant interactions between the polymer chains [26]. In the PPC spectra, the stretching vibration peaks at 3320 cm^−1^, 1749 cm^−1^, and 1225 cm^−1^ were attributed to the O-H, C=O, and C-O-C stretching vibrations in the PPC, respectively. Compared to the composite film with PPC, the O-H peak of PPC was significantly weakened, indicating that the carboxyl group and hydroxyl group of PPC reacted with the epoxy group of ADR. The intensity of the C=O absorption peak of the composite film increased after the addition of PPC, and the absorption band of the composite film was slightly blue-shifted compared to that of PPC because the carbonyl group of PPC could form hydrogen bonds in the composite film and change its vibration frequency. The chemical structure of each component of the composite film is shown in Figure 2. The presence of epoxy groups in the ADR chain extender could act as a molecular bridge between the components to cross-link them, and the possible molecular binding mechanism of each component of the composite film could be derived by combining FTIR analysis.

The tensile strength and elongation at the break of the composite film are shown in Figure 1b. The tensile strength of the composite film increased and then decreased as the proportion factor of PPC in the composite film increased, and when the proportion factor of PPC was 30%, the tensile strength of the composite film was 92.15 MPa, which was 19.68% higher than that of the composite film without PPC. The tensile strength of the composite film decreased to 70.05 MPa at a 40% PPC addition, which is 9.03% lower than the tensile strength of the composite film without PPC addition. And with the increasing amount of PPC introduced, the elongation at the break of the composite films kept increasing, indicating that they had better ductility. This was due to the introduction of PPC, and the copolymer in the composite film was located at the two-phase interface, which reduced the interfacial tension, improved the interfacial compatibility, and increased the bonding force.

**Figure 3:**
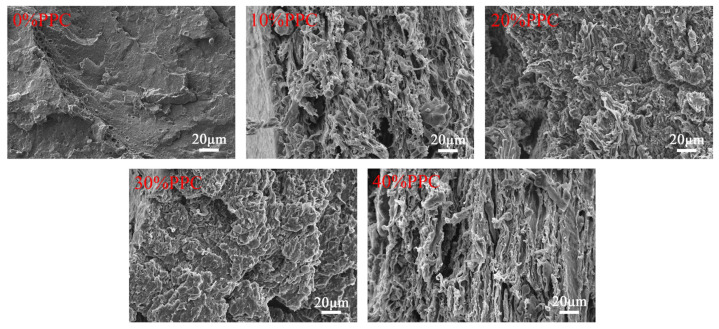
SEM of microscopic morphology of composite film sections.

As shown in Figure 3, the fracture surface of the composite film displayed an island-type structure with unevenness, which is a typical ductile fracture surface. In contrast, the fracture surface of the composite film without PPC was flatter and more uniform. Small spherical particles were visible in the cross-section of the composite films containing PPC, and the interface between the particles and the matrix was clear because the intermolecular interaction between PBAT and PPC was maintained by the entanglement of polymer molecular chains. When PPC with a scale factor of no more than 30% was introduced, it was observed in the cross-section of the composite film that PPC was dispersed in the PLA matrix as a dispersed phase in the form of spherical particles, showing the typical “island structure” of the dispersed phase with a good interface. The addition of PPC improved the compatibility of PLA and PBAT, resulting in stronger interfacial adhesion and reduced phase separation, with the composite film showing good homogeneity. Due to the improved compatibility, the interfacial bond between the two phases was enhanced, which helped improve the mechanical properties of the composite film. The surface roughness and irregularity of the fracture cross-section of the composite film increased when the addition of the ratio of PPC was 40%. It was difficult to obtain a flat cross-section, which was not a completely homogeneous spherical dispersion morphology. The morphology changed from the typical “island structure” of the dispersed phase to that characterized by a bicontinuous phase. This could be further confirmed by the fact that when the high content of PPC was added to the composite film, it resulted in PPC agglomeration, which led to a significant decrease in the tensile strength of the composite film.

**Figure 4:**
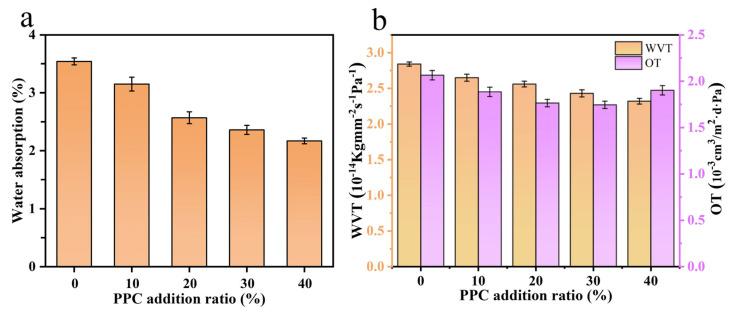
Saturation water absorption of composite films (**a**) and water vapor and oxygen transmission rate of composite films (**b**).

As shown in Figure 4a, the water absorption of the composite film gradually decreased as the proportion of PPC introduced increased, and the decrease in water absorption was due to the addition of PPC to enhance the interaction and adhesion between each polymer, and it was more difficult for water molecules to penetrate inside the composite film structure. The lower water absorption of the laminate film facilitated the laminate film to package the food in daily life, thus improving the application range of the laminate film [27]. The water vapor and oxygen transmission rate coefficients of the composite film are shown in Figure 4b. The water vapor transmission rate coefficient of the composite film without PPC was 2.84, and the water vapor transmission rate coefficient of the composite film was gradually reduced from 2.65 to 2.32 when the introduction ratio coefficient of PPC was gradually increased from 10% to 40%. The introduction of PPC improved the water vapor barrier of the laminate film, which, in turn, made it more suitable for use in food packaging. As can be seen from Figure 4b, the oxygen transmission coefficient of the composite film without PPC was 2.065, and the addition of PPC reduced the oxygen transmission coefficient of the composite film. When the proportion of PPC was introduced at a factor greater than 30%, the oxygen transmission coefficient of the composite film reached a minimum value of 1.745 and remained basically unchanged, which was approximately 18.34% lower than that of the composite film without PPC. The results of the oxygen transmission coefficient indicated that the addition of a certain amount of PPC increased the ability of the composite film to block oxygen, which was related to a reduction in the free volume of the polymer in the composite film. When a certain amount of PPC was added, the interaction and adhesion between the phases increased, reducing the free volume and increasing the resistance to gas diffusion in the film [28], but too much PPC caused phase separation and, thus, the oxygen transmission coefficient of the composite film increased slightly again, at which point the oxygen transmission coefficient was still lower than that of the composite film without PPC.

### 2.2. Thermal Analysis of Laminated Films

**Figure 5:**
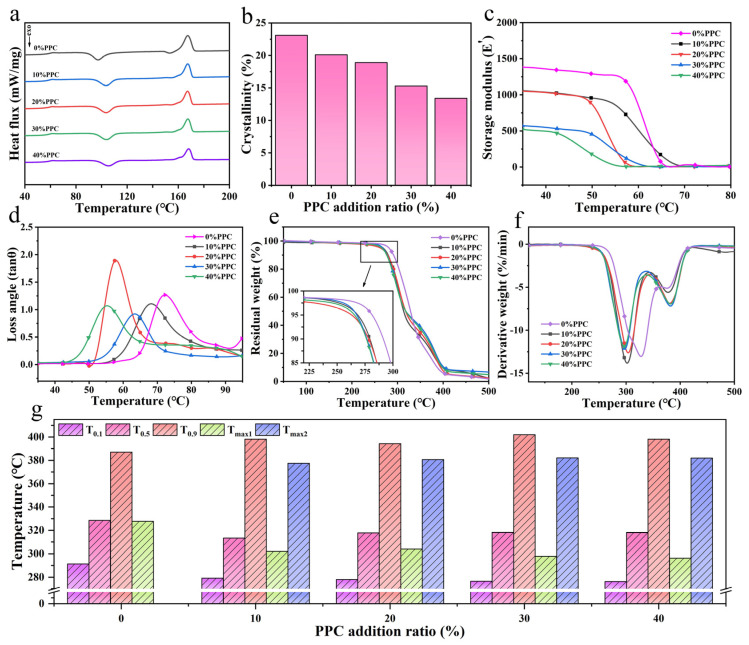
DSC curves of composite films (**a**), crystallinity of composite films (**b**), energy storage modulus (**c**) and loss angle (**d**) of composite films, TG (**e**) and DTG (**f**) curves of composite films, with thermal stability variation in composite films (**g**).

The DSC secondary temperature rise curves and parameters of the composite films are shown in Figure 5a and Table 1. The glass transition temperature (T_g_) of the composite film increased from 61.4 °C to 62.1 °C with the increase in the PPC content. The increase in T_g_ was attributed to the presence of PPC in the composite film, which enhanced the structural stability of the molecular chains and increased the free energy barrier for the chain segment movement to some extent. As the PPC content increased, the cold crystallization temperature (T_c_) of the composite film gradually increased from 96.9 °C to 104.8 °C. The composite film could be cooled and shaped at a higher temperature, thus accelerating the solidification process. It can also be seen from Figure 5b that the crystallinity (X_c_) of the composite film decreased with the increase in the PPC content from 23.1% to 13.4%, and the final crystallization state was determined by both the nucleation process and the crystal growth process, while the introduction of PPC causing the energy barrier of chain segment movement to become higher during the nucleation process of the composite film, and the regular arrangement of molecular chains received influence so that the crystal growth process in the composite film was restricted [29].

The energy storage modulus (E′) and loss angle (tan θ) curves for the composite film are shown in Figure 5c,d, with each storage modulus decreasing with increasing temperature. The energy storage modulus of the composite film was 1049 MPa when the PPC was introduced at a scale factor of 10% and declined rapidly at 56.2 °C. As the amount of PPC introduced increased, the E′ of the composite film decreased, and the temperature corresponding to the decrease also decreased. The loss factor of the composite film with different levels of PPC also showed a peak with increasing temperature, with the peak of the loss angle of the composite film moving towards lower temperatures as the PPC content increased.

Figure 5e,f show the TG and DTG curves of the composite film. The change in thermal stability is shown in Figure 5g, where the T_0.1_, T_0.5_, T_0.9_, and T_max_ values corresponded to the temperature at which the weight loss of the composite film is 10%, 50%, 90%, and the fastest rate of weight loss, respectively. With the increase in the PPC content, T_0.1_ and T_0.5_ decreased, and T_0.9_ increased. The introduction of PPC increased the temperature range of the mass reduction in the composite film. When the introduction coefficient of the PPC ratio increased to 30% and 40%, the T_0.9_ of the composite film increased from 387 °C to 402 °C and 398.2 °C, respectively. The increase in T_0.9_ was due to the hydrogen bond between the carbonyl group of PPC and the hydroxyl group in the film, and the structure of the composite film was more stable. We found that the composite film exhibited a two-stage decomposition process. The maximum degradation temperature in the first stage (T_max1_) and the maximum degradation temperature in the second stage (T_max2_) corresponded to the irregular fracture of PPC and the random chain break of PBAT. The addition of PPC reduced the T_max1_ of the composite film, and T_max1_ decreased with the increase in the PPC content in the composite film, which was related to the destruction of hydrogen bonds between PPC and PBAT and the formation of more terminal hydroxyl groups when the temperature rose. Because the terminal hydroxyl groups induced the degradation of PPC, the hydroxyl groups formed in the blend intensified the thermal degradation. However, due to the entanglement of molecular chains in the composite film, T_max2_ increased with the introduction of PPC, and the TG curve of the composite film effectively transferred to the high-temperature region. The above results show that the addition of PPC improved the thermal stability of the composite film and had a positive impact on its application range.

### 2.3. Composting Degradation and Bacteriostasis Analysis of Composite Film

The composting degradation method was used to study the degradability of the composite film. The value of the composting degradation mass percentage of the composite films with days is shown in Table 2. Compared to the composite film without PPC, the mass reduction percentage of the composite film with PPC was greater than that without PPC at the same time as composting degradation, which gradually increased with the increase in the PPC content.

**Figure 6:**
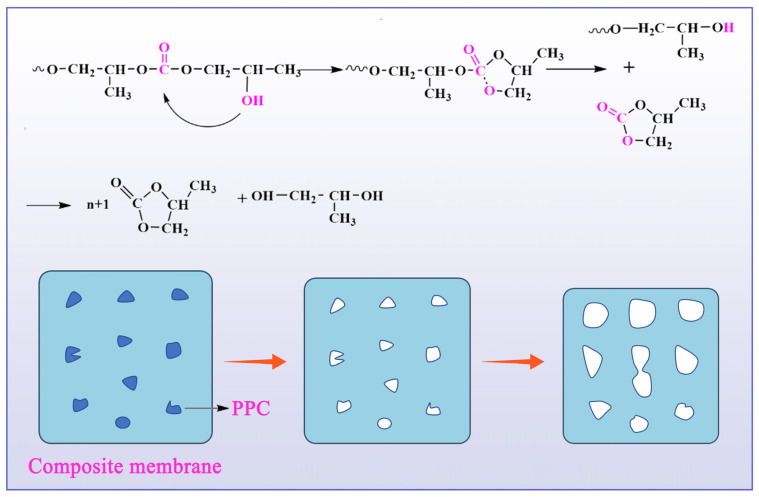
Unzipping degradation of PPC.

The degradation methods of polymer materials are mainly divided into main chain degradation to produce oligomers and monomers, side chain hydrolysis to produce soluble main chain polymers, and cross-chain point cracking to produce soluble polymers. The degradation of the composite film was bulk degradation; that is, the main chain was degraded to produce oligomers and monomers for degradation. During the composting process, water molecules attack the ester bond in the polymer molecular chain, making the ester bond decompose into carboxyl and hydroxyl groups, which is the reverse reaction of the esterification reaction. Degradation consists of the following four processes: composite film water absorption, ester bond breaking, oligomer diffusion, and dissolution. Water molecules contacted the surface of the PLA film and slowly diffused into the vicinity of ester bonds or hydrophilic groups in the molecules. The ester bonds in PLA and PBAT molecular chains in the composite film began to break, and the quality of the composite film slowly decreased. PPC can undergo unzipping degradation, which belongs to the autocatalytic process of hydroxyl groups at the end of PPC, as shown in Figure 6. With the aggravation of degradation, the number of oligomers increased. The diffusion rate of water-soluble, low-molecular-weight degradation products in the surface layer of the composite film and the substrate was inconsistent. The degradation products were easy to diffuse into the composting environment. The carboxyl group and the buffer in the composting degradation solution were neutralized; therefore, the degradation rate of the surface layer of the composite film was reduced.

**Figure 7:**
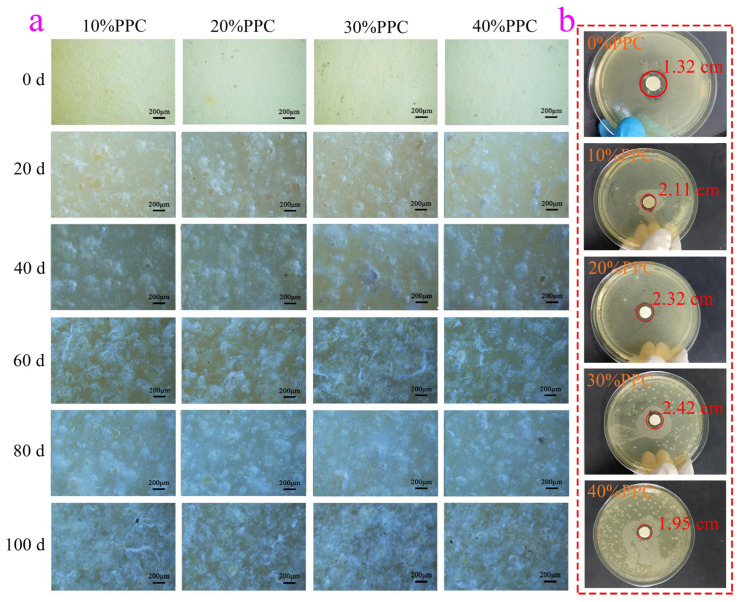
Compost degradability (**a**) and antibacterial properties (**b**) of composite films.

It can be seen from Figure 7a that when the degradation was not started, the surface morphology of the composite film was smooth without clear change. After 20 days of degradation, the mass loss of the composite film containing PPC was not much at 5%–6%. This was because the composite film first absorbed the water molecules produced by composting materials and began to degrade. The water molecules contacted the surface of the composite film and slowly diffused into the vicinity of the ester bond or hydrophilic group in the molecule. The mass loss of the composite film increased with the content of PPC in the composite film. It was observed that the surface of the composite film began to whiten slightly, and a few holes appeared on its surface. After 40 days of degradation, the mass loss of the composite film containing PPC began to increase gradually, which was due to a large number of ester bond breaks in the composite film, resulting in a significant reduction in the quality of about 18%. The degradation rate in this cycle was 200% faster than that in the previous cycle. At this time, a large area of white spots and a large number of holes were observed on the surface of the composite film. After 60 days of degradation, the mass of degradation loss of the composite films containing PPC was 33–37%, and the degradation rate of the composite film in this cycle was about 50% higher than that in the previous cycle. The surface of the composite film was rough, with a large number of uneven holes and a small number of cracks. This was because the degradation of PPC in the composite film was an autocatalytic process of PPC terminal hydroxyl after a large number of ester bonds in the composite film were broken. With the extension of degradation time, the number of terminal hydroxyl groups of PPC increased, and the diffusion rate of water-soluble low molecular weight degradation products in the surface layer of the composite film and a consistent substrate, which was also the reason why the degradation rate of the composite film containing PPC was higher than that of the composite film without PPC. After 80 days of degradation, the degradation quality of PPC containing the composite film reached about 50%, and the degradation rate of the composite film was almost stable. The surface of the composite film was rough, and a large number of uneven holes were connected into gullies. After 100 days of degradation, the degradation quality of the composite film containing PPC reached 65%–70%, and the degradation rate of the composite film was stable compared to that of the previous degradation cycle. A large number of uneven holes formed in the degradation process were densely arranged on the surface of the composite film, and clear cracks appeared in the deep gully, indicating that the degradation process was stable at this time.

Figure 7b shows the antibacterial effect of the composite film on *Staphylococcus aureus*. It was found that there were antibacterial circles around the composite film, the bacterial colonies around the sample were significantly reduced, and the propagation of bacteria was inhibited. The composite film showed certain antibacterial properties against *Staphylococcus aureus*. Compared with the composite film without PPC, the antibacterial effect of the composite film with PPC was better. When the proportion coefficient of PPC was 30%, the antibacterial performance of the composite film was the best, and the diameter of the antibacterial circle could reach 2.42 cm.

## 3. Materials and Methods

### 3.1. Materials

The PPC, chain extender ADR (food grade 4468), and PBAT were produced by BASF, Berlin, Germany. PLA (4032D) was produced by Nature Work, New York City, NY, USA. CS (98% deacetylation) was produced by Shanghai Aladdin Biochemical Technology Co., Ltd., Shanghai, China.

### 3.2. Preparation of Composite Films

PLA, PPC, and PBAT were dried in a constant temperature oven (DGG9070, Shanghai Yiheng Instrument Co., Ltd., Shanghai, China) at 80 °C for 12 h; CS was dried in a constant temperature oven at 105 °C for 12 h; and the food grade epoxy chain extender ADR was dried in a constant temperature oven at 60 °C for 8 h. After the raw materials were dried, they were weighed and manually well-mixed in the following ratios shown in Table 3. Then, the materials were melt-blended by a twin-screw extruder (SHJ20, Nanjing Jainte Mechatronics Co., Ltd., Nanjing, China) with a temperature setting of 135 °C-160 °C-180 °C-180 °C-135 °C in each area, and the blended PLA/PPC/PBAT/CS/ADR mixture was crushed in the multi-functional crusher. Finally, the obtained crushed mixture was hot-pressed for 5 min under a 180 °C hot press (BL6170, Dongguan Baolun Precision Instruments Co., Ltd., Dongguan, China) with a pressure setting of 8 MPa to obtain composite films of 100 mm × 100 mm × 0.2 mm.

### 3.3. Characterization

#### 3.3.1. Fourier Transform Infrared Spectroscopy Analysis

The chemical structure of PLA-based composite films was characterized by Fourier transform infrared spectroscopy (FTIR, tensor, Bruker, Bremen, Germany), and its wave number range was 500–4000 cm^−1^. 

#### 3.3.2. Mechanical Properties Test

According to GB1040.3-2006 [30], the tensile test of the composite film was carried out with a mechanical testing machine (CMT5504, Shenzhen Xinsansi Testing Co., Ltd., Shenzhen, China). The tensile speed was set to 5 mm/min, and each group of samples was tested seven times in parallel. The maximum and minimum values were removed, and the average value and deviation were calculated as the measurement results. 

#### 3.3.3. Scanning Electron Microscopic Analysis

A scanning electron microscope (SEM, QUANTA 220, FEI, Hillsboro, OR, USA) was used to observe the cross-sectional morphology and structure of the PLA-based composite film, and the samples were tested after gold spray treatment.

#### 3.3.4. Water Absorption Test

According to GB1034-98 [31], the PLA-based composite film was cut into three 25 mm × 25 mm sizes, placed in a drying oven at 60 °C for 72 h, and then removed and weighed as W_1_. It was soaked in a beaker with 300 mL of distilled water, removed and wiped off the surface every 72 h, and weighed again as W_2_. The average value was calculated and recorded, and after one month of immersion, the composite films reached saturated water absorption, and the water absorption (WA) was calculated according to Equation (1).

(1) $$\mathrm{WA}={(W}_{2}-W_{1})/W_{1}\times 100\%$$

#### 3.3.5. Water Vapor Permeability Test

According to the requirements of ASTME96-1995 [32], the thickness of the PLA-based composite film (recorded as X) was measured with a vernier caliper and 2 g of anhydrous calcium chloride was added to the test glass bottle, at which time the relative humidity (recorded as RH_2_) of the test glass bottle was 0. The test glass bottle was sealed with the PLA-based composite film and then weighed, and the glass bottle was placed in a desiccator with a saturated NaCl solution at the bottom (at which time the temperature was kept at 25 °C and the relative humidity was kept at 75%), and at which time the relative humidity in the desiccator was recorded as RH_1_. We placed the desiccator into a thermostat with the temperature set at 25 °C. The glass bottle mass was weighed and recorded at intervals of 72 h using a balance, and the change in the glass bottle’s mass (m)–time (T) was plotted as a function of the slope of the curve k in the function plot. The water vapor transmission rate (WVTR) was equal to the calculation of the slope of the curve in the function graph k divided by the glass bottle mouth area, which is the test temperature (25 °C) under the saturation vapor pressure (P) of water vapor. Water vapor permeability (WVP) is shown in Equation (2).

(2) $$\mathrm{WVP}=\frac{\mathrm{WVTR}}{P(\mathrm{RH}1-\;\mathrm{RH}2)}X$$

#### 3.3.6. Oxygen Permeability Test

The oxygen permeability meter (OX2/231, Languang Instruments Co., Ltd., Jinan, China) was used to test each group of three parallel samples simultaneously on the oxygen permeability meter machine according to the requirements of GB/T 19789-2021 [33]. After vacuuming for 3 h, the oxygen permeability test was conducted, and the data were recorded.

#### 3.3.7. Differential Scanning Calorimetry Test

The thermal properties of PLA-based composite films were examined by differential scanning calorimetry (DSC, Diamod DSC, Perkin-Elmer, Waltham, MA, USA). The heating rate was set to 10 °C/min from 20 °C to 200 °C and kept constant at 200 °C for 10 min, then cooled down from 200 °C to −20 °C with the cooling rate set to 10 °C/min, and then from 20 °C to 200 °C with the heating rate set to 10 °C/min. The secondary heating curves were analyzed and calculated by Equation (3) for their crystallinity.

(3) $$X_{c}={(\Delta H}_{m}-{\Delta H}_{\mathrm{cc}})/{(\Delta H}_{f}\times W_{\mathrm{PLA}})\times 100\%$$

where ∆H_cc_ is the enthalpy of cold crystallization, ∆H_m_ is the enthalpy of melting, ∆H_f_ is the enthalpy of melting when PLA is fully crystallized with a value of 93.7 J/g, and W_PLA_ is the percentage of PLA in the composite film [34].

#### 3.3.8. Dynamic Thermomechanical Analysis

Dynamic thermomechanical analysis (DMA, NETZSCH, Hanau, Germany) tests were performed on a DMA242 equipped with a stretching fixture, where the PLA-based composite film was warmed from 35 °C to 100 °C, with the ramp rate set at 10 °C/min and the frequency set at 1 Hz.

#### 3.3.9. Thermogravimetric Analysis

A thermogravimetric analyzer (TGA, 209F3, NETZSCH, Germany) was used to test the thermal stability of the composite film. Weighing 5 mg of the dried sample (upper and lower deviations were about 0.5 mg), the purge gas used was argon, the temperature was increased from 30 °C to 500 °C, the heating rate was set to 10 °C/min, and the thermal stability analysis was performed by retaining the curve.

#### 3.3.10. Compost Degradability Analysis

According to the requirements of GB/T16716.7-2012 [35], the test samples were cut into 3 parts of 50 mm × 10 mm in size, which were parallel for each group, with the mass weighed, recorded as m_1_, and placed into the compost bucket. (The composting environment consisted of three daily foods, three rotting fruits and composted bran, and the composting environment remained stable for 100 days). The samples were taken out every 20 days, the surface was cleaned, the test mass was recorded as m_2_, the weight loss rate of the composite film compost degradation was calculated, and the average value was calculated and recorded. The surface morphology of the composite was recorded using a polarized light microscope (OLYMPUS, Beijing Ruike Zhongyi Technology Co., Ltd., Beijing, China). The weight loss rate (W_d_) of compost degradation was calculated according to Equation (4).

(4) $$W_{d}={(m}_{1}-m_{2})/m_{1}\times 100\%$$

#### 3.3.11. Antibacterial Properties Test

In accordance with the GB/T31402-2015 [36] standard, the bacterial inhibition performance of the composite film was tested by cutting the samples into 10 mm diameter discs and making 3 parallel samples for each group. The samples were sterilized in an electric pressure steam sterilizer at 103 kPa and 120 °C for 20 min and then removed to an ultra-clean bench. *Staphylococcus aureus* was aseptically inoculated in tryptic soy broth (TSB) and incubated at 37 °C for 20 h. The inoculum was then aseptically inoculated onto a new TSB Petri dish, and samples were added and incubated at 37 °C for 24 h. Finally, the circle of inhibition was measured to document the inhibitory effect of the composite films.

## 4. Conclusions

To enrich the properties of PLA-based composite films and improve their degradability, PPC was introduced to form a complementary relationship with PLA. Firstly, with the introduction of PPC, the mechanical properties of the composite film were improved, and the tensile strength of the composite film was 92.15 MPa, which was 19.68% higher than that of the composite film without PPC. Secondly, the addition of PPC reduced the water vapor permeability coefficient and oxygen permeability coefficient, and the antibacterial property of the composite film was enhanced, which had a positive significance for the composite film used in food packaging. Thirdly, PPC improved the crystallization ability of the composite film. The PPC phase can be used as a heterogeneous nucleation point to promote the nucleation of molecular chains in the composite film, which is conducive to the processing and molding of the composite film. Eventually, for the compost degradability of composite films, the degradation rate increased with the increase in the PPC content. It is important for the recycling treatment of the composite film after disposal, which is conducive to the protection of the ecological environment.

## Figures and Tables

**Table 1:** DSC data for composite film.

PPC Addition Ratio	T_g_/°C	T_c_/°C	T_m_/°C	∆H_c_/(J/g)	∆H_m_/(J/g)	X_c_/%
0	61.4	96.9	166.9	15.4	29.9	23.1
10%	61.6	102.8	167.1	15.9	27.2	20.1
20%	61.7	103.2	167.2	16.3	25.7	18.9
30%	61.9	104.2	167.6	16.5	23.2	15.3
40%	62.1	104.8	167.9	17.1	22.1	13.4

**Table 2:** Composting degradation weight loss percentage of composite film.

PPC Addition Ratio	0 Days	20 Days	40 Days	60 Days	80 Days	100 Days
0	0	4.69%	12.17%	23.47%	35.39%	46.58%
10%	0	5.49%	17.54%	33.76%	48.96%	63.9%
20%	0	5.96%	18.03%	34.74%	49.95%	64.76%
30%	0	6.37%	18.52%	36.4%	53.38%	69.93%
40%	0	6.59%	18.86%	37.23%	54.7%	71.57%

**Table 3:** Raw material ratio of composite film.

PPC Addition Ratio (PPC/(PLA + PPC))	0	10%	20%	30%	40%
PLA	66.53 wt%	59.88 wt%	53.22 wt%	46.57 wt%	39.92 wt%
PPC	0	6.65 wt%	13.31 wt%	19.96 wt%	26.61 wt%
PBAT	28.51 wt%	28.51 wt%	28.51 wt%	28.51 wt%	28.51 wt%
CS	3.96 wt%	3.96 wt%	3.96 wt%	3.96 wt%	3.96 wt%
ADR	1 wt%	1 wt%	1 wt%	1 wt%	1 wt%

## Data Availability

Data are contained within the article.
